# Tarsal tunnel syndrome in the mucopolysaccharidoses: A case series and literature review

**DOI:** 10.1002/jmd2.12021

**Published:** 2019-03-14

**Authors:** Nicole Williams, Jake Willet, Damian Clark, David Ketteridge

**Affiliations:** ^1^ Department of Orthopaedic Surgery Women's and Children's Hospital Adelaide Australia; ^2^ Centre for Orthopaedic and Trauma Research University of Adelaide Adelaide Australia; ^3^ Training Medical Officer Unit, Royal Adelaide Hospital Adelaide Australia; ^4^ Department of Neurology Women's and Children's Hospital Adelaide Australia; ^5^ Department of Genetics and Molecular Pathology Women's and Children's Hospital Adelaide Australia

**Keywords:** decompression, surgical, genetic diseases, inborn, mucopolysaccharidoses, orthopaedics, paediatrics, tarsal tunnel syndrome

## Abstract

**Background:**

The mucopolysaccharidoses (MPS) are a group of inherited, progressive, multi‐system lysosomal storage disorders. Musculoskeletal manifestations include nerve entrapment syndromes, most commonly carpal tunnel syndrome. Tarsal tunnel syndrome (TTS) has also been reported. The purpose of this study was to investigate the clinical course of MPS patients with suspected TTS and to conduct a literature review of TTS in MPS.

**Methods:**

A review of the Medline and EMBASE databases was conducted in accordance with published guidelines from the Joanna Briggs Institute of Evidence Based Medicine with search strategy developed by a librarian trained in systematic reviews. A medical record review was undertaken for all patients managed in the multi‐disciplinary MPS clinic in a tertiary referral paediatric centre, identifying patients with a suspected or established diagnosis of TTS. Data regarding the demographics, investigations, presentation, management, and clinical course were collected.

**Results:**

The literature review failed to identify any published papers regarding TTS in MPS, with conference proceedings only identified. Within a cohort of 19 MPS patients, four patients with a suspected diagnosis of TTS were identified (MPS I: two patients, MPS VI: two patients). Three patients underwent surgical tarsal tunnel decompression, two with good result. One patient had overlapping symptoms with spinal stenosis and improvement in suspected tarsal tunnel symptoms following spinal decompression and fusion.

## 1 INTRODUCTION

The mucopolysaccharidoses (MPS) are a group of inherited, progressive, multi‐system lysosomal storage disorders. Eleven enzyme deficiencies result in seven MPS types. Clinical manifestations reflect insufficient glycosaminoglycan degradation, with resultant breakdown product deposition within many tissues and secondary metabolic effects.^1^

Enzyme replacement therapy (ERT) or haematopoietic stem cell transplant (HSCT) prolong lifespan and improve quality of life for certain MPS patients. However many MPS musculoskeletal complications remain largely unimproved.^2^

**Figure 1:**
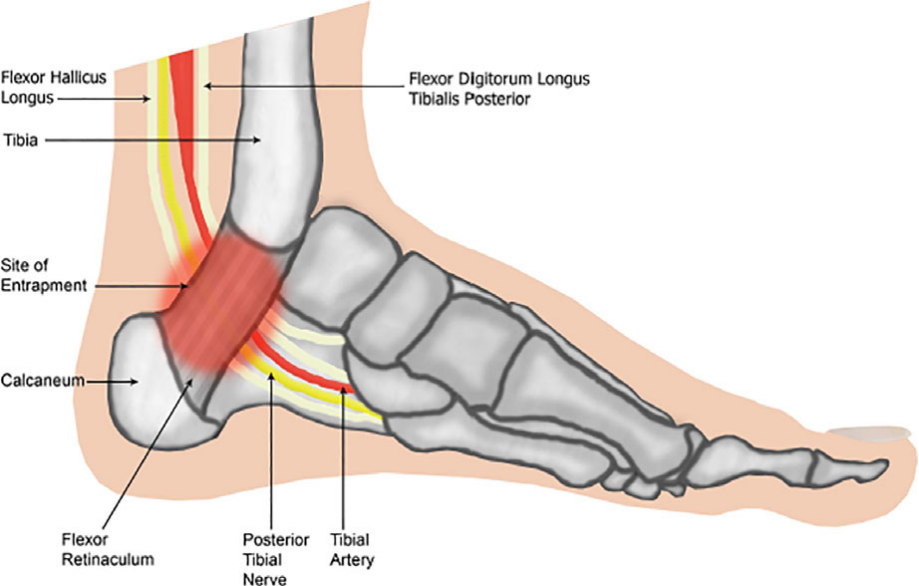
The Tarsal Tunnel—illustration of the tarsal tunnel with underlying structures; the tibial nerve, posterior tibial artery and tendons of the tibialis posterior, flexor digitorum longus and flexor hallicus longus muscles, passing deep to the flexor retinaculum

The most common nerve entrapment syndrome in MPS is the median nerve at the carpal tunnel and MPS is the most common cause of carpal tunnel syndrome (CTS) in childhood.^3^ CTS occurs frequently in MPS I (Hurler syndrome), MPS II (Hunter syndrome), and MPS VI (Maroteaux‐Lamy syndrome) and is rare in MPS IV (Morquio syndrome).^4^ Routine neurophysiological CTS screening is recommended to allow early surgical decompression and prevent permanent impairment.^5^ Other nerve entrapment syndromes occur less commonly in MPS,^6^ including tarsal tunnel syndrome (TTS): compression of the posterior tibial nerve as it passes inferiorly to the flexor‐retinaculum within its fibro‐osseous tunnel^7^ (Figure 1).

We report our experience with diagnosis and management of TTS in MPS patients seen in a tertiary referral paediatric hospital and present the results of a scoping review of the scientific literature regarding TTS in MPS.

## 2 METHODS

### 2.1 Literature review

A scoping literature review was conducted in accordance with published guidelines from the Joanna Briggs Institute of Evidence Based Medicine (2015)^8^ in order to determine the breadth of scientific literature pertaining to TTS in MPS, with the aim to progress to systematic review if sufficient available evidence. The search strategy was developed with assistance from a medical librarian with expertise in systematic reviews. Medline and EMBASE online databases were searched via the University of Adelaide hosted by Ex Libris Primo, using combinations of relevant keyword and subject headings (see Supplemental Material). English language articles only were reviewed with publication dates: Medline 1946 to 5 May 2018, EMBASE 1974 to 5 May 2018. Articles were initially screened using title and abstract. Two reviewers (NW and JW) made all decisions regarding article inclusion, initially independently and later together to achieve consensus in the case of any discrepancies.

### 2.2 Case reports

Institutional ethics committee approval was obtained for retrospective medical record review (Audit No 644A) for all patients with a diagnosis of MPS seen by the metabolic service at a tertiary referral paediatric hospital. Patients were identified from the metabolic service patient database and excluded if the only contact with the service was in the context of an enzyme therapy replacement trial, with regular multi‐disciplinary care at another institution. In patients with a suspected or established diagnosis of TTS, the following information was extracted from the medical record: gender, MPS type, MPS treatment (ERT and/or HSCT), age at TTS diagnosis, TTS signs and symptoms, investigations, treatment, treatment complications and findings at most recent follow‐up.

## 3 RESULTS

### 3.1 Literature review

**Figure 2:**
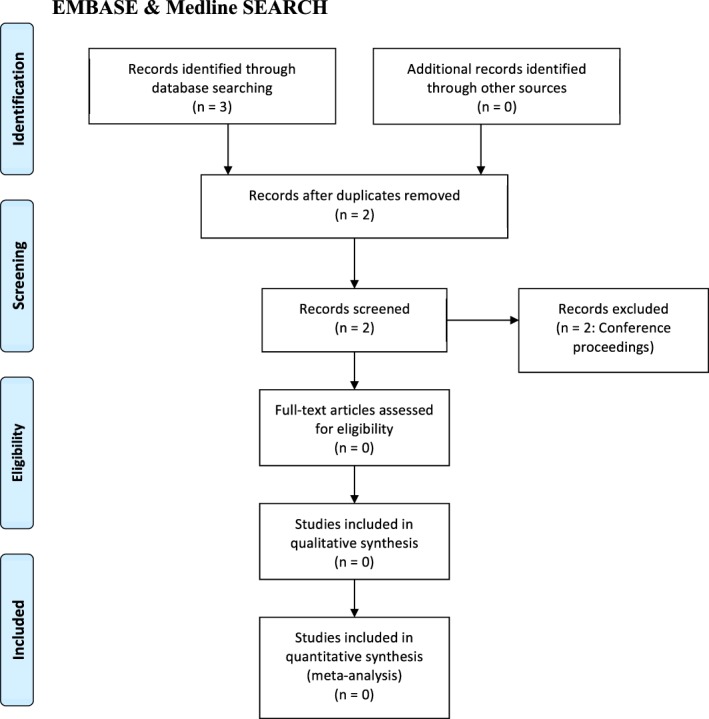
PRISMA flow diagram—PRISMA flow diagram showing the results of a combined Medline and EMBASE search and subsequent article review^11^

The initial Medline and EMBASE searches identified three articles. Following initial title and abstract screening, one repeat was excluded and the full‐text was retrieved for the remaining two articles. Both were conference proceedings. One described two MPS VI patients with TTS^9^ and the other reported TTS in their inclusion criteria but only reported patients with CTS.^10^ Therefore, the review failed to identify any published scientific articles describing TTS in MPS patients. The PRISMA flow diagram details the search results (Figure 2).

### 3.2 Case reports

Medical records for 19 MPS patients: MPS I (Hurler syndrome), 6 patients; MPS III (San Fillipo syndrome), 6 patients; MPS IV (Morquio A syndrome), 2 patients; and MPS VI (Maroteaux‐Lamy syndrome), 5 patients were reviewed. Four patients with a suspected diagnosis of TTS were identified (MPS I, 2 patients; MPS VI, 2 patients).

#### 3.2.1 Case 1: Female, MPS VI

This patient, aged 6.5 years, who was not receiving systemic therapy for MPS, presented with a 3‐week history of bilateral hand and foot pain and persistently rubbing her hands and feet. A clinical diagnosis of bilateral CTS and TTS was made and bilateral carpal tunnel and tarsal tunnel decompressions were performed the following month.

The operative report described a longitudinal incision from just distal to the medial malleolus to just distal to the medial process of the navicular. The neurovascular bundle was identified. The flexor retinaculum and Master Knot of Henry were released. The patient was observed in the paediatric intensive care unit overnight and discharged home after a two nights of hospital stay.

A right foot wound dehiscence 4 days postoperatively required her return to operating room for re‐suturing. At 6 weeks postoperatively, the patient was asymptomatic and no recurrence of symptoms was documented in the medical record. The patient experienced increasing complications of her MPS condition over the ensuing years. She commenced ERT at 9.5 years of age with some systemic improvement, but she passed away at age 16 years following complications of bowel obstruction.

#### 3.2.2 Case 2: Male, MPS VI

This patient also had no systemic MPS treatment. At 8 years of age, he complained of occasional tingling in hands and feet. Nerve conduction studies (NCS) suggested motor and sensory deficits in the median nerve at the carpal tunnel. The lateral popliteal nerves demonstrated normal conduction velocities.

Bilateral carpal tunnel release and right tarsal tunnel release were performed. The tarsal tunnel surgery involved a longitudinal incision along the adductor margin. The retinaculum was divided at the Master Knot of Henry. No obvious plantar nerve compression was noted although the connective tissues were thickened. The left side was not decompressed as a control in view of the pre‐operative neurophysiological testing findings. Minor otorhinolaryngological procedures were performed under the same general anaesthetic. Discharge from hospital was delayed for 5 days as the patient lived far from the hospital and also developed diarrhoea post‐operatively.

No appreciable improvement in symptoms followed unilateral tarsal tunnel release and the left side was not decompressed. The patient's walking became increasingly limited, and he went on to have bilateral femoral osteotomies when he was 10 years old. He later underwent HSCT, at age 12, and remains alive at age 34.

#### 3.2.3 Case 3: Female, MPS I

This patient underwent HSCT at 31 months of age. She, at age 9 years and 9 months, developed a tingling sensation initially in the left foot and was referred to a neurologist for investigation. She had previously undergone successful bilateral carpal tunnel surgery. NCS of the lower limbs were inconclusive, with normal distal tibial motor responses and electromyography (EMG) of abductor hallucis brevis (AHB) muscle, suggesting chronic neuropathic changes with large polyphasic units seen. She, at age 10 years and 5 months, was referred to an orthopaedic surgeon for consideration of tarsal tunnel release. Concurrent investigations by the spinal team for multi‐level spinal stenosis identified significant compression at the thoracolumbar junction in association with a large pathological vertebral disc. A recent decrease in walking distance was attributed to myelopathy by the spinal surgeon. The decision was made to defer any consideration of tarsal tunnel surgery until after spinal surgery. Posterior spinal decompression and fusion was performed. At 5 months following spinal surgery, the patient's walking distance and tingling in the feet had significantly improved.

#### 3.2.4 Case 4: Male, MPS I

This patient, aged 13 months, underwent HSCT. He, at age 13 years, underwent carpal tunnel surgery, following pain and decreased hand function. NCS for CTS were inconclusive. Post‐operatively, there was resolution of pain and increased hand function. Pyridoxine‐responsive homocystinuria was diagnosed at age 15 and he began oral pyridoxine therapy with good response. At age 16, he developed painful feet, similar in character to his hand pain prior to carpal tunnel surgery. He was reluctant to wear shoes secondary to the pain, would only wear slippers and would often rub his feet and had increasing refusal to walk. NCS were inconclusive, with normal distal tibial motor responses and some large polyphasic units on EMG of AHB. The neurologist's opinion was that the symptoms were consistent with a nerve compression syndrome. Bilateral tarsal tunnel release was performed at age 17 years and 10 months.

The operative report described a medial incision. The posterior tibial nerve was identified posterior to the medial malleolus and then explored distally. Both medial and lateral plantar nerves were identified and released. Tenosynovitis was debrided. Standing transfers were permitted post‐operatively. He underwent ophthalmological and dental examinations under the same general anaesthetic. He was discharged home the day following surgery and described complete resolution of symptoms at 6 week follow‐up.

## 4 DISCUSSION

Nerve compression syndromes in MPS may result from multiple factors, including deposition of GAG breakdown products within connective tissue structures and abnormal bony development with deformity.^12^, ^13^ Foot and ankle deformities that may contribute to posterior tibial nerve compression in MPS include ankle and hindfoot valgus, equinus, and forefoot adductus with prominence of the first metatarsal head.^14^ Despite increasing utilisation of life‐prolonging systemic therapies, including HSCT, particularly in MPS I and ERT for MPS I, II, IV‐A and VI, many musculoskeletal complaints, including CTS, remain uncorrected in MPS patients, and orthopaedic surgery is probably increasing in this population.^2^

TTS has been reported in MPS,^9^ yet our literature review failed to identify a single scientific paper regarding TTS in MPS. This is likely due to its rarity, but it is possible that TTS is underdiagnosed in MPS. TTS is frequently misdiagnosed in non‐MPS patients^7^ as a result of subjective and inconsistent history and examination findings.^15^ Patients may describe vague symptoms of foot pain, and paraesthesia, often described as worst at night or during prolonged standing.^7^, ^16^ Examination may reveal sensory changes over terminal branches of the posterior tibial nerve and tenderness on deep palpation,^16^ atrophy of the intrinsic foot muscles and a positive Tinel's sign, which in CTS has a sensitivity and specificity of 82.2% and 88.9%, respectively.^17^ The dorsiflexion‐eversion test may assist in TTS diagnosis and is performed by passively maximally everting and dorsiflexing the ankle, while all of the metatarsophalangeal joints are maximally dorsiflexed. This position is held for 5 to 10 seconds with intensification of symptoms experienced.^15^ Thenar atrophy and anhidrosis are seen in MPS patients with CTS.^4^ Muscle wasting and/or anhidrosis were not documented for any of our patients. The two patients in our series who complained of significant foot pain and rubbing their feet pre‐operatively had the best results post‐operatively with elimination of pain in both cases. The case with tingling only had minimal change post‐operatively.

Reasons for failure to diagnose CTS in MPS may include masking disease features such as joint stiffness and skeletal dysplasia, prioritisation of more pressing complaints, cognitive impairment and inability to cooperate in sensory testing.^4^, ^18^ Many patients deny symptoms despite florid signs and severe neurophysiological deficits.^4^ Similar factors may mask the TTS diagnosis in MPS patients. In one MPS I patient in our series, it was difficult to determine whether altered sensation and pain in the lower limb were due to posterior tibial nerve pathology or nerve root compression at the spinal level. The decision was made to proceed initially with spinal decompression as this pathology was considered more clinically significant.

Neurophysiological testing can be utilised in adjunct to diagnose nerve compression. For CTS in MPS, screening with regular NCS is recommended, although there is currently no standardised screening regime.^19^ Screening is recommended as many MPS patients will not present with clinical signs and symptoms of CTS and early surgical decompression may minimise permanent neurological damage.^3^, ^4^, ^18^, ^20^ Neurophysiological testing for CTS is stated to have a specificity of 80% and sensitivity 89% in the non‐MPS population.^21^ In TTS, increased latencies and slowing of conduction across the flexor retinaculum may be observed; however, NCS have a high false negative rate, and a review failed to determine the actual sensitivity and specificity, negating their role in excluding pathology.^22^, ^23^ Neurophysiological testing in compression neuropathies is particularly difficult in bilateral disease, such as MPS, with no ‘normal’ side for comparison. In our series, one patient had excellent symptomatic relief and return of function following tarsal tunnel release, despite non‐confirmatory neurophysiological testing pre‐operatively. Another patient had a good surgical result when surgery was performed based on clinical findings without neurophysiological testing. A third patient had non‐confirmatory neurophysiological tests and no improvement in symptoms after decompression. A fourth did not undergo surgery, despite suggestive nerve conduction studies, due to the presence of clinically significant spinal cord pathology. Based on our small experience, we would not recommend routine neurophysiological testing for TTS in MPS patients, but would consider it in equivocal cases, being aware of the potential for false negatives. We have not used injection with corticosteroid and local anaesthetic as a diagnostic or treatment modality for TTS in MPS, but this is described in the non‐MPS population and could be considered.^7^, ^16^, ^24^

Ultrasound has been used in the non‐MPS population to identify space occupying lesions within the TTS and sensitivities of up to 74% have been reported for diagnosis of idiopathic TTS.^25^, ^26^ There is no literature regarding ultrasound for assessment of TTS in MPS and we have not used this modality for this purpose. Interpretation may be difficult in this population due to lack of experience.

In non‐MPS patients, initial management of TTS usually involves non‐surgical treatments with anti‐inflammatory medication, physiotherapy and shoe modification.^7^, ^16^, ^24^ Success rates following surgical decompression vary from 44% to 96%.^16^ A narrative review of TTS in non‐MPS patients recommends early decompression in selected patients in order to prevent nerve fibrosis.^16^ There is insufficient evidence to direct management decisions for MPS patients with TTS. Within the MPS literature, non‐surgical management of CTS is not generally recommended, with early decompression considered advisable to prevent progression and permit functional improvement.^3^, ^4^, ^18^, ^20^, ^27^ There is insufficient evidence to advise for or against early surgery in suspected TTS in MPS. One of our patients experienced 12 months of symptoms, including severely limited function prior to decompression with complete symptomatic relief after surgery.

We treated patients with MPS I and MPS VI for TTS. As these MPS types are associated with prominent musculoskeletal involvement, including widespread skeletal dysplasia (dysostosis multiplex),^28^ it can be assumed that TTS would occur most commonly in these types, as well as MPS II and less commonly in MPS IV, where joint instability predominates and nerve compression syndromes are rare and also MPS III, which is characterised by prominent neurocognitive decline.^29^

MPS patients have increased risk of surgical and anaesthetic complications^28^, ^29^ and some high‐risk surgical procedures may be performed in MPS patients without evidence of improvement in quality of life and function.^28^, ^29^, ^30^ The only surgical complication documented in this series was a wound dehiscence requiring re‐suturing under general anaesthetic. Surgical benefit with symptomatic relief was seen in two patients, with no benefit of surgery in a third. However, this is a very small series. TTS may be considered as a cause of foot pain in MPS patients, and may manifest as persistently rubbing the feet. Surgical release can be considered particularly if impacting upon daily activities or quality of life. Surgeons treating MPS patients should be encouraged to share their experiences. Prospective evaluation of function and quality of life before and after surgery will add to our understanding of the role of surgery for these patients.

## CONFLICTS OF INTEREST

A/Prof Nicole Williams has received honoraria, travel and travel expenses to attend educational medical symposia and research grants from BioMarin Pharmaceutical Inc.

Dr Jake Willett and Dr Damian Clark declare that they have no conflict of interest.

Dr David Ketteridge has received honoraria and travel expenses to attend educational medical symposia from BioMarin Pharmaceutical Inc.

## AUTHOR CONTRIBUTIONS

A/Prof Nicole Williams was involved in project design, case note review and preparation of case reports, literature review, manuscript preparation and submission and study guarantor. Dr Jake Willet was involved in literature review and first draft manuscript preparation. Dr Damian Clark contributed the Specialist Paediatric Neurology content, including neurophysiology study interpretation and relevant literature synthesis. Dr David Ketteridge was involved in study concept, contributed to the case reports and manuscript review.

## Supporting information


**Appendix 1:** Medline Search (1946 to May 2018)
**Appendix 2:** EMBASE search (1974 to May 2018)
