# Supplementary material for: Tarsal tunnel syndrome in the mucopolysaccharidoses: A case series and literature review
Source: JIMD Rep. 2019 Mar 14;46(1):16–22. doi: 10.1002/jmd2.12021 (PMC6498821; doi:10.1002/jmd2.12021)
Supplement: Supplementary file 1 — Appendix 1: Medline Search (1946 to May 2018) Appendix 2: EMBASE search (1974 to May 2018) [file JMD2-46-16-s001.docx]

**Appendix 1 :** Medline Search (1946 to May 2018)

| **Mucopolysaccharidoses** | **Tarsal Tunnel Syndrome** |
| --- | --- |
| exp MUCOPOLYSACCHARIDOSES(6046)  OR  mucopolysaccharidosis.mp. (5824)  OR  mps.mp. (9225)  OR  lipochondrodystrophy.mp. (399)  OR  hunter syndrome.mp. or  Mucopolysaccharidosis II/ (1022)  OR  exp Mucopolysaccharidosis I/ (1675)  OR  hurler syndrome.mp. (428)  OR  sly syndrome.mp. or exp Mucopolysaccharidosis VII/ (276)  OR  exp Mucopolysaccharidosis VI/ or maroteaux lamy.mp. or exp Mucopolysaccharidosis IV/ (1622)  OR  exp Mucopolysaccharidosis III/ (627)  (14105) | tibial nerve compression.mp. (20)  OR  tibial tunnel syndrome.mp. (0)  OR  exp Tibial Neuropathy/ (665)  OR  exp Tarsal Tunnel Syndrome/ (538)  OR  tibial nerve entrapment.mp. (16)  (682) |

Medline search identified 0 articles as at May 2018

**Appendix 2:** EMBASE search (1974 to May 2018)

| **Mucoplysaccharidoses** | **Tarsal Tunnel Syndrome** |
| --- | --- |
| mucopolysaccharidoses.mp. or exp mucopolysaccharidosis/ (11283)  OR  MPS.mp. (14366)  OR  lipochondrodystrophy.mp. or exp Hurler syndrome/ (2744)  OR  exp mucopolysaccharidosis type 7/ or exp Hunter syndrome/ or mucopolysaccharidosis I.mp. (2596)  OR  mucopolysaccharidosis II.mp. (231)  OR  mucopolysaccharidosis III.mp. or exp Sanfilippo syndrome/ (1151)  OR  mucopolysaccharidosis IV.mp. or exp Morquio syndrome/ (1398)  OR  mucopolysaccharidosis V.mp. (3)  OR  mucopolysaccharidosis VI.mp. or exp Maroteaux Lamy syndrome/ (1003)  OR  exp Hunter syndrome/ or mucopolysaccharidosis VIII.mp. (1841)  OR  sly syndrome.mp. (88)  OR  mucopolysaccharidosis VII.mp. (157)  (22181) | exp tarsal tunnel syndrome/ or tibial nerve entrapment.mp. (1016)  OR  tibial nerve compression.mp. (27)  OR  tibial tunnel syndrome.mp. (0)  OR  tibial neuropathy.mp. (44)  OR  (tibial adj2 syndrome).mp. [mp=title, abstract, heading word, drug trade name, original title, device manufacturer, drug manufacturer, device trade name, keyword, floating subheading word, candidate term word] (951)  (1981) |

EMBASE search identified 3 articles as at May 2018
